# Implementing interventions to reduce antibiotic use: a qualitative study in high-prescribing practices

**DOI:** 10.1186/s12875-021-01371-6

**Published:** 2021-01-23

**Authors:** Aleksandra J. Borek, Anne Campbell, Elle Dent, Christopher C. Butler, Alison Holmes, Michael Moore, A. Sarah Walker, Monsey McLeod, Sarah Tonkin-Crine, on behalf of the STEP-UP study team, Philip E. Anyanwu, Aleksandra J. Borek, Nicole Bright, James Buchanan, Christopher C. Butler, Anne Campbell, Ceire Costelloe, Benedict Hayhoe, Alison Holmes, Susan Hopkins, Azeem Majeed, Monsey McLeod, Michael Moore, Liz Morrell, Koen B. Pouwels, Julie V. Robotham, Laurence S. J. Roope, Sarah Tonkin-Crine, Ann Sarah Walker, Sarah Wordsworth, Carla Wright, Sara Yadav, Anna Zalevski.

**Affiliations:** 1 Nuffield Department of Primary Care Health Sciences, University of Oxford, Radcliffe Observatory Quarter, Woodstock Road, Oxford, OX2 6GG UK; 2 National Institute for Health Research (NIHR) Health Protection Research Unit in Healthcare Associated Infections and Antimicrobial Resistance, Imperial College London, London, UK; 3 Primary Care Population Sciences and Medical Education, Faculty of Medicine, University of Southampton, Southampton, UK; 4 NIHR Health Protection Research Unit in Healthcare Associated Infections and Antimicrobial Resistance, University of Oxford, Oxford, UK; 5 NIHR Oxford Biomedical Research Centre, Oxford, UK; 6 Nuffield Department of Medicine, University of Oxford, Oxford, UK; 7 Centre for Medication Safety and Service Quality, Pharmacy Department, Imperial College Healthcare NHS Trust, London, UK; 8 NIHR Imperial Patient Safety Translational Research Centre, Imperial College London, London, UK

**Keywords:** General practice, Antibiotic resistance, Antimicrobial stewardship, Antibiotics, Back-up prescription, Point-of-care testing, Focus groups

## Abstract

**Background:**

Trials have shown that delayed antibiotic prescriptions (DPs) and point-of-care C-Reactive Protein testing (POC-CRPT) are effective in reducing antibiotic use in general practice, but these were not typically implemented in high-prescribing practices. We aimed to explore views of professionals from high-prescribing practices about uptake and implementation of DPs and POC-CRPT to reduce antibiotic use.

**Methods:**

This was a qualitative focus group study in English general practices. The highest antibiotic prescribing practices in the West Midlands were invited to participate. Clinical and non-clinical professionals attended focus groups co-facilitated by two researchers. Focus groups were audio-recorded, transcribed verbatim and analysed thematically.

**Results:**

Nine practices (50 professionals) participated. Four main themes were identified. Compatibility of strategies with clinical roles and experience – participants viewed the strategies as having limited value as ‘clinical tools’, perceiving them as useful only in ‘rare’ instances of clinical uncertainty and/or for those less experienced. Strategies as ‘social tools’ – participants perceived the strategies as helpful for negotiating treatment decisions and educating patients, particularly those expecting antibiotics. Ambiguities – participants perceived ambiguities around when they should be used, and about their impact on antibiotic use. Influence of context – various other situational and practical issues were raised with implementing the strategies.

**Conclusions:**

High-prescribing practices do not view DPs and POC-CRPT as sufficiently useful ‘clinical tools’ in a way which corresponds to the current policy approach advocating their use to reduce clinical uncertainty and improve antimicrobial stewardship. Instead, policy attention should focus on how these strategies may instead be used as ‘social tools’ to reduce unnecessary antibiotic use. Attention should also focus on the many ambiguities (concerns and questions) about, and contextual barriers to, using these strategies that need addressing to support wider and more consistent implementation.

**Supplementary Information:**

The online version contains supplementary material available at 10.1186/s12875-021-01371-6.

## Background

Mitigating the spread of antimicrobial resistance by more prudent antibiotic use is a public health priority. Most antibiotics are prescribed in general practice (72% in 2018) [1], largely for respiratory tract infections (RTIs) which are often self-limiting [2, 3]. England has seen a gradual reduction in antibiotic prescribing but with significant variation in prescribing rates within and between practices, even after accounting for factors such as comorbidities and deprivation [2–5]. Moreover, there is wide variation in prescribing to less unwell patients [6]. It is now important to identify ways to facilitate (further) optimisation of antibiotic prescribing in practices that have remained high-prescribing.

Considerable evidence supports various interventions to safely reduce antibiotic prescribing for RTIs [7–9]. Among them are delayed (back-up, deferred) antibiotic prescriptions (henceforth DPs) and point-of-care C-Reactive Protein testing (POC-CRPT) (Table 1). Trial evidence shows that DPs can help safely reduce antibiotic use for acute RTIs, with only 33–39% of patients given DPs using antibiotics [10–13]. The National Institute for Health and Care Excellence (NICE) recommends considering DPs for selected common infections [14, 15]. Similarly, trial evidence shows that POC-CRPT helps safely reduce antibiotic prescribing [16] (e.g., by 15% in acute cough [17] and by 22% for chronic obstructive pulmonary disease exacerbations [18] compared to usual care). NICE supports using POC-CRPT in adults with cough [19]. However, both interventions have not been routinely implemented in UK general practice, and the influences on uptake, implementation and effectiveness of these strategies outside of clinical trials have not been adequately researched.

**Table 1:** Definitions of DPs and POC-CRPT and related guidelines

**Delayed (also referred to as back-up or deferred) antibiotic prescriptions (DPs)**	
The NICE defines DP as a prescription “given in a way to delay the use of [the antibiotic], and with advice to only use it if symptoms worsen or don’t improve within a specified time. The prescription may be given during the consultation (which may be a post-dated prescription) or left at an agreed location for collection at a later date​”. (NICE Glossary) NICE guidance recommends considering DPs for: • acute cough in patients with higher risk of complications, • acute sore throat (with FeverPAIN scores of 2 or more or Centor score 3–4), • acute otitis media (unless systemically very unwell or high risk of complications), • sinusitis if there is no improvement for more than 10 days (unless systemically very unwell or high risk of complications), • lower urinary tract infections for non-pregnant women. (Summary of antimicrobial prescribing guidance – managing common infections; updated in March 2020, available on: https://www.nice.org.uk/Media/Default/About/what-we-do/NICE-guidance/antimicrobial%20guidance/summary-antimicrobial-prescribing-guidance.pdf)	
**Point-of-care C-Reactive Protein Testing (POC-CRPT)**	
C-reactive protein (CRP) is a marker of inflammation that increases 4–6 h after the onset of inflammation or acute tissue injury and peaks at 36–90 h (1.5–4 days). Various point-of-care CRP tests are available that require a small blood sample from a finger prick and that produce a quantitative or semi-quantitative result within approx. 3 to 10 min. NICE clinical guideline [CG191] supports the use of POC-CRPT to help differentiate a serious infection from a self-limiting RTI in adults with acute cough (lower RTI) when, after clinical assessment, a diagnosis of pneumonia has not been made. It suggests interpreting POC-CRPT results as follows: • CRP < 20 mg/l: no routine antibiotic • CRP between 20 and 100 mg/l: delayed/back-up antibiotic • CRP > 100 mg/l: immediate antibiotic.	

Many qualitative studies have explored clinicians’ views on antibiotic stewardship interventions, but often as process evaluations within clinical trials [20–24]. These identified that general practitioners (GPs) prefer multifaceted interventions which decrease diagnostic uncertainty, help provide patient-centred care and are easy to implement [25], and that they experience interventions as ‘supportive aids’ but also potentially as a compromise, source of distress, or unnecessary [26]. Few qualitative studies have focussed on implementing DPs [27, 28] and POC-CRPT [29–31] in the UK outside of trials. One study found prescribers used DPs infrequently, mainly to avoid anticipated conflict and because of feeling uncomfortable with burdening patients with clinical responsibility, and perceiving DPs as a conflicting message [28]. Another study found DP use was influenced by: GPs’ prior experiences of using DPs, views on how to protect the doctor-patient relationship, lack of agreed prescribing strategies within and between practices, and lack of feedback on how DP affects antibiotic prescribing data [27]. Studies found the implementation of POC-CRPT as influenced by: cost/reimbursement, time, effect on workload and flow, access to equipment, physical and operational constraints, quality control and training, practitioner attitudes and experiences, local champions, and gaps in evidence [29–31].

This study aimed to explore the views of professionals in high-prescribing general practices on use and implementation of DPs and POC-CRPT. In doing so, we build on, and extend, previous studies by identifying how these two strategies could be implemented to optimise antibiotics in the ‘real world’ outside clinical trials and in practices that remain high-prescribing despite other initiatives used to reduce prescribing (e.g. national data monitoring and targets). We also specifically sought to explore views and suggestions to guide implementation and uptake of both strategies for commissioners, practices and/or clinicians who may be considering using them.

## Methods

### Participants

We identified general practices which were in the top 20% for antibiotic prescribing in the West Midlands Clinical Research Network (CRN), based on 2017 PrescQIPP data (antibiotic items per STAR-PU (Specific Therapeutic group Age-sex Related Prescribing Unit)) [32]. We used the antibiotic items per STAR-PU as it is commonly used in England to compare antibiotic prescribing across practices and against prescribing targets. Study invitations were sent to 139 practices and then followed-up by email and/or phone. Additionally, the study was promoted by CRN Facilitators. Interested practices were asked to identify suitable date(s) for a focus group with at least three professionals (comprising at least two prescribers and any other clinical or administrative staff). Participants gave written consent at the start of focus groups. Practices were offered £500 reimbursement for one focus group.

### Data collection

We collected data through focus groups in participating practices to facilitate discussions among professionals and elicit shared as well as divergent views on the use of the antibiotic optimisation strategies and practice-level implementation (rather than only individual use). Focus groups took place in general practices between December 2018 and April 2019. They were facilitated by two researchers (AB, AC) – each leading a part of the focus group, while the other made notes. Discussions followed a semi-structured topic guide (see Additional file 1) which was piloted with three GPs. The topics included: making antibiotic prescribing decisions, experiences of using DPs, views on POC-CRPT (with three types of tests shown to prompt discussion), practice communication and other antibiotic stewardship strategies used. As DPs are used by prescribers (although variably), we explored participants’ experiences; as POC-CRPT is rarely available in UK practices, we explored views about hypothetical use. Focus groups were audio-recorded and transcribed verbatim. Transcripts were anonymised, checked for accuracy with audio-recordings, and speakers’ professional roles were added based on notes.

### Data analysis

Data were analysed inductively using thematic analysis [33] with coding in NVivo software (v.12). We used thematic analysis because it is a systematic qualitative data analysis method, suitable for applied health research, and allows the analysis to be driven by the data (by inductive coding) as well as the development of interpretations (themes) that extend understanding beyond just a summary of data. Initially four researchers (AB, AC, STC, ED) coded the same 2–3 transcripts, discussed coding and categories for the initial codes, and agreed on a coding framework. The coding framework was then used by AC and AB to code all remaining transcripts independently, adding new codes when needed, and then combining their analyses. Themes were identified, discussed and agreed with a multidisciplinary team (AB, AC, STC, MM, ED), and then reviewed by the wider study team (including GPs, epidemiologists and behavioural economists).

## Results

Nine practices participated, including 50 professionals (3–11 per practice) (Table 2). Focus groups lasted 49–87 (mean 71) minutes. No practice had used POC-CRPT, although two (FG2, FG3) had the equipment. Four main themes were identified; additional quotes are available in Additional File 2.

**Table 2:** Practice characteristics

Focus group	Urban / rural	Deprivation (decile)^a^	FG participants ^**b**^
FG1	Rural (village)	Medium (5)	2 GPs, Nurse, HCA, Practice Manager
FG2	Rural (town and fringe)	Medium (5)	GP, Pharmacist Prescriber, Business Partner
FG3	Urban (major conurbation)	High (2)	2 GPs, Prescribing Clerk, Practice Manager
FG4	Rural (town and fringe)	Medium (4)	2 GPs, Nurse, Practice Manager
FG5	Rural (town and fringe)	Low (7)	3 GPs, 2 GP Trainees, Nurse Prescriber, Practice Manager
FG6	Urban (major conurbation)	High (3)	4 GPs, Medicines Coordinator
FG7	Urban (major conurbation)	High (2)	3 GPs, Nurse
FG8	Urban (major conurbation)	High (2)	6 GPs, 1 GP Trainee, 2 Nurses, Practice Manager, Deputy Practice Manager
FG9	Urban (major conurbation)	High (1)	2 GPs, 2 GP Trainees, HCA, 2 Receptionists

^*a*^ *Index of multiple deprivation decile.* ^*b*^ *GP – General Practitioner, HCA – Healthcare Assistant (non-prescriber), Nurse – Practice Nurse (non-prescriber)*

### Compatibility of strategies with clinical role and experience

Participants’ views on DPs and POC-CRPT were influenced by their perceptions on how these strategies fit with their clinical role and experience. They reported how the core clinical role in general practice (developed through training and experience) involved clinical assessment based on history-taking, examination and social factors. The clinical assessment could be also informed by, but prioritised over, clinical scores (e.g. Centor, FeverPAIN) and diagnostic tests.

DPs and POC-CRPT were described as fitting with the clinical role and useful when there is clinical uncertainty over diagnosis or prognosis, such that DPs could provide a safety-net and POC-CRPT additional clinical information. Such uncertainty was described as quite rare, though this depended on the experience of the prescriber.

*When I use a deferred script it’s normally because I feel it’s a bit more of a borderline case… a patient where you’re not entirely sure and so it’s there for the patient if they worsen… [GP, FG2]*

*Those people that are right on the fence, where you’re uhming and ahhing… it’s quite rare for a doctor not to know what type of infection you’ve got. [GP, FG4]*

GPs described their roles as ‘holistically’ ‘*treating patients, not numbers*’ [FG1] and that POC-CRPT would *unlikely* add much to, or change, their clinical judgment if not uncertain. In contrast with secondary care, participants highlighted that diagnostic testing was not routine in general practice.

*A lot of your training in primary care is diagnosing patients without test interpretation. If you go into hospital, you get a battery of tests (…) It makes you more reliant on test results… Then the more of these things are used I think maybe it does somehow take away from the clinicians… [Manager: Art.] Yes, it may take [away] some of your clinical judgement. [GP, FG2]*

Clinical experience seemed to influence perceptions of usefulness of DPs and POC-CRPT. Some GP trainees reported using DPs more (one accounted it to lower confidence in clinical decisions); with more experienced GPs reporting using DPs less frequently, preferring immediate or no prescription. GPs and nurses described POC-CRPT as likely to be used more by trainees, and GPs perceived POC-CRPT as more helpful for nurses and pharmacists who may rely more on test results to reduce clinical uncertainty. More experienced clinicians described feeling more confident using their clinical judgment irrespective of tests. They were concerned that dependence on POC-CRPT by trainees might lead to loss of clinical skills.

*It’s difficult at the start of training in that you’ve not got that much experience and (…) you’re more worried about making a mistake. I probably had a bigger range of ones that were in the middle and (…) felt more comfortable having that safety-net [of DPs]. [GP Trainee, FG5]*

*GP1: I think [GP trainees would] test everybody… Because you give them any equipment and they use it religiously… they don’t look at the patient…*

*GP2: I think it would probably make them less clinical.*

*GP1: They’re so reliant now on the machines and the templates… tick, tick, tick, do the test and then treat. You haven’t actually looked at your patient yet. [FG3]*

### Strategies used as social tools to negotiate treatment and educate patients

Participants frequently described (perceived) patient expectations for antibiotics as a driver for unnecessary antibiotic use; some described reducing antibiotic prescriptions as beyond their control.

*Antimicrobial resistance is beyond the surgery’s control a lot of the time because it is patient expectations (…) the patient insists and insists and that’s not the clinician’s fault that antibiotics are prescribed in the end. [GP, FG2]*

They discussed using DPs and POC-CRPT as social tools to negotiate treatment with patients perceived as difficult to reassure when not needing antibiotics (‘*regular returners*’ [FG6], ‘*frequent offenders*’ [FG8]). Most GPs reported using DPs as a compromise when they considered antibiotics unnecessary but felt that patients wanted antibiotics; a GP trainee [FG5] described how with increased experience he used DPs less as a safety-net and more often as a compromise. Participants also envisaged using POC-CRPT as ‘evidence’ to convince patients when antibiotics are unnecessary and ‘*deny patients antibiotics… more than deciding on antibiotics*’ [GP, FG9]. Both strategies were seen as helping avoid lengthy negotiations, conflict, complaints and re-consultations; and helping maintain good relationships, patient satisfaction and more patient-centeredness (‘*equal footing within the consultation*’ [FG7]). They were also seen as strategies to educate patients that antibiotics are unnecessary.

*GP1: I tend to use [DP] in the people you just cannot convince that they don’t need antibiotics. (…) sometimes it’s just the route of least resistance…*

*GP2: …you’re using it as a trade-off… saying, ‘come on, give my way a bit of a chance, let’s see how it goes’… and if then in a few days they’re starting to feel a little bit better, they say, ‘okay, we’re on the right track’, and that’s when they don’t come in for the antibiotics. (…) I suppose it leads to improved patient satisfaction, because they feel they’re not being fobbed off… it’s the key to not getting complaints. [FG4]*

Participants described patients as expecting a prescription and preferring tests and numbers, and some reported already using clinical scores or tests to negotiate treatment decisions (Table 3). Others felt that patients were accepting of no-antibiotic decisions and reassurance with effective communication. Participants were also concerned that using POC-CRPT may have unintended consequences, such as unexpectedly high test results, raising patients’ expectations for tests and ‘*medicalising*’ common infections.

*It’s funny, the amount of times that you’re advised to treat the patient not the number, the patient will be much happier with the number than your clinical judgement. [GP, FG2]*

*Nurse: Here it’s a small population and it’ll get around the patients and they’ll say, ‘well why did they have that test and I didn’t’?*

*GP: Yeah… so then you’ll end up having to do it. [FG4]*

**Table 3:** Using ‘social tools’ to help address perceived patient expectations

**• Perceived that patients expect to leave ‘with something’ – use of prescriptions and leaflets**	
I have a very simple rule… They’ve made the effort to come and see a doctor, give them a bit of advice, or even a **prescription** or a **form for physio** or something like that, it’s the key to not getting complaints. **Everybody gets a prize**, even if it’s just a **bit of written paper**. [GP, FG4]	
I like to print out a post-dated prescription because actually giving them **something in their hand** to go away with gives them **a sense that something’s happening**. [GP, FG6]	
…patients are used to have **something to take away** with them, so when they come they need something... whether it is **a prescription**... Sometimes what may help is on EMIS you’ve got **patient information leaflets**…[GP, FG7]	
They tend to like to **leave with something** and if it’s not antibiotics and not what they want, they seem to want to leave with **some form of prescription** be it an **over the counter medicine** or be it something else... I’m seeing more requests for things like nasal sprays and linctus… [GP, FG9]	
**• Perceived that patients want tests and numbers – use of POC tests**	
It’s funny, the amount of times that you’re advised to treat the patient not the number, the patient will be much **happier with the number than your clinical judgement**. [GP, FG2]	
I could also use [POC-CRPT] on these frequent offenders who come in saying ‘I want antibiotics’… if you show them it’s not this and **it’s not 100 and that convinces them** in some ways psychologically not to get the antibiotic. [GP, FG8]	
A really good thing is to have a tool to demonstrate to patients why they don’t need antibiotics. I **use my SATS probe** quite a lot as a... it’s not really a tool but it helps me, I’ll kind of say ‘Well your oxygen saturations are very good.’ Which is why I’m very interested in testing CRP ‘cause I think that’s a really good evidence based tool, which if patients understand, are going to be more receptive and accepting of your decision not to give them antibiotics, if you can actually **demonstrate numerically** that there’s no reason to. [GP, FG6]	
…like **urine samples**, they come in for urine symptoms when you think ‘This is not’ – and then you dip it and say ‘Look, there’s none.’ But that’s cheap… [GP, FG6]	
If you got a printout, you can give them a copy, it’s a prize, they’ve had a test… They think tests are how we do medicine, and they’re not… ‘oh, I **need a test, I need a scan’**. [GP, FG4]	
**• Perceived that patients need ‘evidence’ – use of clinical scores**	
I use the **FeverPAIN** to not give them antibiotics because it’s just – it’s helpful to be like ‘well **the computer says you don’t need them!**’ And sometimes that works [laughter] better than ‘the doctor says you don’t need them!’ …it does unfortunately bite me in the bottom sometimes when they come back again and say: ‘well what does your score say?’ And it comes out saying ‘you need a delayed prescription!’ So they go away with a delayed prescription when probably clinically I wouldn’t have given them anything at all. [GP, FG3]	
Sometimes what can help in sore throat is the **Centor or the FeverPAIN**, so you can actually show them the scoring criteria and say, ‘X, Y and Z, because you haven’t got any of those criteria, **evidence shows** us that it’s very unlikely that this is bacterial and this is in fact viral.’ [GP, FG7]	

### Ambiguities about usefulness and impact of strategies

Participants considered pros and cons of DPs and POC-CRPT, and situations and patients when these strategies should or should not be used, with apparent ambiguity and contradictions. DPs were seen as helping relieve patients’ anxiety by improving access to antibiotics (e.g., before weekends) while reducing the need to re-consult. Some prescribers reported using DPs for adults and children with RTIs and patients with additional risks (e.g. immuno-compromised); in contrast, others (sometimes the same participants) reported *not* using DPs for adults with RTIs (preferring to either prescribe or not), children (preferring to re-consult) or at-risk patients (preferring to prescribe or re-consult).

*If it’s for a child, then I’d rather review them. If you have a compromised patient or a diabetic patient, then I might issue the script because I know they are at a higher risk so it all depends. It’s not a fixed thing. It just depends on the individual. [GP, FG8]*

Some participants reported using DPs with ‘sensible’ patients – those they perceived to ‘*understand the use of antibiotics*’ [FG8] and ‘*on board with [DP]*’ [FG6] (Table 4). Although no participant described patients as ‘insensible’, some reported concerns about potential intentional or unintentional misuse (i.e. they or others using the antibiotics immediately or in the future without consulting). For these patients, they reported being more likely to add a ‘*second step (…) [or] any slight impediment in their way [so] they won’t use it if they don’t need to*’ [FG4] or ‘*an extra layer of awkwardness*’ [FG5], for example, by leaving the prescription at reception to be collected in a few days or post-dating it.

**Table 4:** Participants reporting using DP with ‘sensible’ patients

I only give delayed antibiotics if I feel like the patient or the patient’s parent is very **sensible** and **on board** with it. [GP, FG6] Generally [DP] is for chest complaints that I would issue it, or if they’ve had recurrent tonsillitis that has required antibiotics that it’s been appropriate for and they’re starting to become unwell and you’ve got a **sensible family**, then I might do it then as well… [GP, FG7] [DP] would be **useful for patients who understand the use of antibiotics, who are bit more sensible but not for everybody** I would say, considering we’ve got some population who doesn’t understand when to use it. Some population groups in this practice who don’t understand when to use antibiotics so they’ll still be feeling okay and will still get antibiotics and take it because they are used to that. [GP, FG8] I also probably **gauge which ones** I think **are more likely to be sensible** hopefully. [Patient’s father] said ‘Yes, I think that sounds reasonable’. He seemed a bit reassured about that. Let’s see how she goes in the next few days. [GP trainee, FG9]	

Participants were unclear about the effectiveness of DPs on reducing inappropriate antibiotic use; some expected DPs to reduce antibiotic use, others thought they may increase it (i.e. when used instead of not prescribing). Participants were also uncertain if DPs count towards prescribing rates if unused. Practices had no set ways of issuing DPs and prescribers discussed with interest what their colleagues did. Prescribers reported choosing DP formats to facilitate ease of antibiotic access if it was helpful (e.g., giving verbal advice to wait when handing a prescription before a weekend/travel) with patients whom they trusted to use DPs appropriately. Conversely, they reported choosing DP formats that deterred patients from using the antibiotic immediately (e.g., by post-dating) when they doubted that patients would use DPs appropriately.

*The biggest disadvantage is that unless it’s post-dated, a proportion of people will go and cash it. That’s what they wanted. [GP, FG5]*

Ambiguity about when and how POC-CRPT should be used was also apparent. Some participants considered whether they could use it when deciding about hospital admission; to monitor recovery over time; to screen and triage patients and for patients with COPD exacerbations. Some participants questioned the usefulness of CRP as a biological marker and the sensitivity and specificity of tests. They were uncertain about interpreting the results (particularly medium values) and how to act on results inconsistent with clinical judgment.

*I want to know what’s the evidence? What kind of infections have they looked at? How do they know if it’s viral or bacterial? I don’t really know. CRP is so non-specific… [GP, FG6]*

Participants had mixed views about the effectiveness of POC-CRPT on antibiotic prescribing. Some envisaged limited impact as they expected it not to change clinical decisions, while some thought it may increase prescribing due to perceived pressure to act on unexpectedly raised test results. Nevertheless, most expressed interest in trying POC-CRPT, and generally thought that it could reduce prescribing associated with perceived patient pressure.

*I would worry that by doing [POC-CRPT] and then getting a result that I wasn’t necessarily expecting, I would then feel obliged to prescribe something because otherwise I’m not acting on an abnormal result. [GP, FG1]*

*If I want to prescribe, I don’t think I’d even do the test. [GP, FG3]*

### Influence of context on use of strategies

Context, including practice characteristics and situational factors, influenced care and use of both strategies (for summary see Table S1 in Additional File 3). High prescribing was felt to be partially a result of practice/staff characteristics (e.g. more locums/trainees and staff turnover) and patient characteristics (e.g. comorbidities, culture/languages, deprivation).

Practice context influenced whether and how DPs were used. Prescribers from practices with ‘good access’ (where patients could get appointments quickly), telephone triage and other available services (e.g., extended access) preferred to re-consult rather than give DPs. Prescribers from rural practices preferred to give DPs, or provide antibiotics from the on-site dispensary, with advice to delay taking antibiotics to minimise patients’ burden of returning to the practice.

*I don’t think we do very many delayed scripts at all because of the way we work, because of the easy access and the dispensary. [GP, FG1]*

Moreover, DPs were described as used less in practices in areas with higher deprivation and patients from certain cultures or non-English speakers (perceived as less likely to use DPs appropriately). The desire to avoid additional workload for the administrative staff and potential conflict with patients made prescribers less likely to ask patients to collect DPs from the practice reception.

Participants raised many practical challenges with implementing POC-CRPT, particularly around time, logistics and cost. GPs considered consultations too short for POC-CRPT and envisaged asking nurses or healthcare assistants to perform the tests. Some considered triaging patients with the tests before their appointment. Participants discussed training and logistical difficulties in storing and maintaining equipment – difficulties they envisaged would disrupt workflows, add workload, and require carefully devised implementation protocols.

*Because it’s so ad-hoc it would be quite difficult for you to know when you’ve got patients and you want to do it and how you’re going to fit it in… in amongst other patients that you’re already seeing, it could be quite tricky. [Nurse, FG4]*

Participants reported limited ability for practices to fund POC-CRPT, seeing additional commissioners’ or government funding as necessary for adoption. Some described how wider contextual influences drove the uptake of POC-CRPT, such as needing to ‘keep up’ with other practices and countries adopting POC-CRPT, and expecting to be increasingly required to use POC-CRPT as evidence for prescribing audits and medico-legal reasons.

*…we’d use it because if everyone else is doing it… and you’re the only one and something goes wrong, then it’s indefensible… In a court of law they’ll say, “well everyone else in the patch is using it, why don’t you use it”? “Because I don’t need to”. “I know, but in this case you were wrong…” What are you going to say then? [GP, FG4]*

## Discussion

Participants reported mixed views about whether or not each strategy would be useful and in what circumstances. Overall, they perceived the strategies to be of limited value as ‘clinical tools’, helpful only in ‘rare’ situations of clinical uncertainty and for less experienced prescribers. By contrast, both strategies were seen as helpful ‘social tools’ to negotiate treatment while maintaining relationships or educating patients that antibiotics may not be necessary, especially for patients perceived to expect antibiotics. However, many prescribers described DPs as a strategy to be used only with ‘sensible’ patients, often choosing the format of DP to make things easier for patients they trusted or create barriers for patients they perceived to expect antibiotics but who did not require them. Participants also reported mixed views and doubts about the perceived impact of DP and POC-CRPT on antibiotic prescribing/use in the real world, outside of trial settings. Participants discussed many contextual and practical issues with implementing DPs and POC-CRPT. There was a prevalent sense of ambiguity and mixed views about the strategies: how they fit in general practice; when and how they should be used; and to what extent the benefits outweigh barriers to implementation.

### Strengths and limitations

We recruited a high number of participants from a relatively diverse range of practices and the nine focus groups provided us with rich data to answer our research question and develop the reported themes and findings. The quality of data collection and analysis was strengthened by involving multiple experienced qualitative researchers and discussions with a multidisciplinary team. The analysis was data-driven and data saturation was achieved, with multiple quotes across all focus groups supporting the findings (see also Additional File 2). The study was reported following relevant standards (with the reporting checklist and additional details in Additional File 3) [34].

Transferability of the findings may be limited as we included high antibiotic prescribing practices from one area in England and some practices had reduced their prescribing rate before the focus group. We used the antibiotic items per STAR-PU as a measure to identify high prescribing practices that may particularly benefit from strategies to support optimising their antibiotic prescribing. High antibiotics/STAR-PU may suggest some suboptimal prescribing but it does not take into consideration potential valid reasons for high prescribing rates such as those practices with high numbers of patients with co-morbidities [5]. In our study, we did not explore in more detail the (in)appropriateness of antibiotic prescribing and only used the antibiotics/STAR-PU as a proxy to identify practices that may have more scope for and benefit from implementing additional strategies to optimise antibiotics. While focus groups allowed participants to discuss and address different views and experiences, the presence of colleagues with different roles might have influenced what individuals shared and led to a dominance of GPs’ views (who tended to speak more). As the practices had not used POC-CRPT, participants’ views were hypothetical and might differ from actual experiences of using POC-CRPT as evidenced previously [23, 24, 35].

### Comparison with existing literature

Similar to existing literature, both strategies were seen as ‘clinical tools’ to help manage clinical uncertainty, especially for those still developing clinical skills/experience: DPs were used to safety-net instead of re-consulting [27, 28, 36] and POC-CRPT to help assess illness severity and whether antibiotics are needed [23, 24, 29, 30, 35, 37–39]. However, we found that clinical uncertainty about RTIs among experienced clinicians was seen as relatively ‘rare’. This resonates with existing literature, with RTI consultations described as ‘simple’ [28]. Other types of uncertainty were apparent. For POC-CRPT, this was not only regarding the quality of tests, but also how results should be interpreted and the perceived pressure to act on results inconsistent with clinical judgement [29, 30, 39] – which were seen to potentially threaten a prescriber’s clinical role and skills. For DP, other types of uncertainty were regarding how patients may use them [28, 36, 40] and how useful DP was as a strategy to reduce inappropriate antibiotic use. Despite clinical guidelines, participants were unclear and had mixed views about when these strategies were clinically suitable. In all, the use of these strategies as ‘clinical tools’ in high-prescribing practices was viewed as limited. This compares with previous studies which found that GPs from low-prescribing practices perceived DPs as more useful as a safety-net than GPs from high-prescribing practices whose use of DP was instead more influenced by social/patient factors [27, 40]. Importantly, it differs in showing that simply providing POC-CRPT equipment or guidelines for use of DPs or POC-CRPT to address ‘clinical uncertainty’ may be insufficient to optimise antibiotic prescribing and prescribers in high-prescribing practices may need to challenge their current ‘confidence’ about prescribing.

Participants seemed more convinced about the usefulness of both strategies as ‘social tools’, especially with patients perceived as expecting antibiotics. Counter to guidance, previous studies also describe clinicians using DPs as a compromise – to maintain relationships, avoid conflict and complaints [27, 28, 40, 41], and to educate patients that antibiotics are not always necessary [10, 27, 41, 42], especially in high-prescribing practices [27]; and POC-CRPT to convince and reassure patients of no need for antibiotics [24, 29, 30, 35, 37–39]. Our participants contrasted *unnecessary* antibiotic prescribing which resulted from perceived patient expectations with *high* antibiotic prescribing arising from contextual factors including patient characteristics and staff/patient turnover. As ‘social tools’, DPs and POC-CRPT were perceived as particularly helpful in high-prescribing practices with higher patient expectations and need for antibiotics. Some participants displayed a paternalistic approach and described their patients as mostly expecting antibiotics, likely to use DPs inappropriately, and that DPs are suitable only for selective (‘*sensible*’) patients [27, 28, 36, 40, 41], and that patients are convinced by tests and numbers. Contrary to their own reservations about POC-CRPT, GPs described stressing the certainty of POC-CRPT to patients. Despite the impact of clinicians’ *perceptions* of patient expectations on prescribing, studies show that these perceptions tend to be overestimated or misjudged [43–45]. Moreover, evidence shows that effective communication skills can help understand and address patient concerns and expectations, maintain good relationships, and educate patients about infections and antibiotics, and may be more sustainable long-term [17, 46, 47].

### Implications

The Covid-19 pandemic has brought forth new uncertainties and challenges for healthcare systems across the world [48, 49], including for prescribers in primary care. Mitigating the spread of antimicrobial resistance by more prudent antibiotic use is an even greater public health priority for pandemic response and preparedness. A first step in managing the new uncertainties around Covid-19 is to identify which uncertainties are already ‘known’ and to minimize these where possible [49].

However, many policies tend to advocate use of DPs and POC-CRPT to only reduce clinical uncertainty. Based on our study findings, this approach lacks sufficient attention to other types of uncertainties and ambiguities relevant to prescribers. It also does not acknowledge nor advise how these strategies may or may not be used as ‘social tools’ in practice. High-prescribing practices *may* benefit from implementing DPs and POC-CRPT but practice staff would first need to be clearer on the benefit of these strategies, in what contexts/situations, and how they would fit with practice. Presenting clinical trial evidence alone is insufficient to motivate intervention adoption. Moreover, it is currently unclear how transferrable the trial evidence is to routine practice in terms of implementation as well as effectiveness of these strategies outside of research contexts. Evaluating these strategies in the ‘real world’ of (high-prescribing) practices would provide a better understanding of whether, when and how these strategies might be useful.

Despite the uncertainties, ambiguities and doubts about these strategies, our participants also perceived them as potentially helpful in certain contexts and situations. As antibiotic prescribing and use are complex behaviours influenced by various determinants, it is unlikely that one or two strategies would ‘solve’ the issue. While DPs and POC-CRPT may be insufficient as stand-alone strategies, they might be useful in addition to other strategies (e.g. audit and feedback, communication skills), or more acceptable to clinicians and/or patients than some strategies (e.g. a no-antibiotic strategy). To support implementation, research should focus on developing and testing implementation strategies; specifically, develop evidence on optimal approaches to implementation; training in when and how to use DPs and POC-CRPT and how to effectively discuss strategies with patients; and investigate (intended and unintended) consequences of using these strategies routinely in high-prescribing practices. Similarly, commissioners and practices/clinicians wanting to increase the use of these strategies need to address the perceived ambiguities about DPs and POC-CRPT and practical challenges; for example, disseminate relevant evidence and guidelines; fund POC-CRPT equipment; help problem-solve practical challenges to use; provide feedback on how patients use DPs; and develop practice-specific protocols for using these strategies consistently.

## Conclusions

In conclusion, our findings extend current knowledge regarding how DPs and POC-CRPT are used as ‘clinical tools’ or ‘social tools’ in UK/English general practice and in other countries [23, 24, 35, 36, 38–41]. They highlight the ambiguities and complexities which teams in high-prescribing practices consider when thinking about implementing these strategies and their impact on antibiotic prescribing/use. Most notably, they explain why high-prescribing practices may not value such strategies as ‘clinical tools’ and thus have important implications for policy advocating for all prescribing to be based on testing [50].

## Supplementary Information

**Additional file 1:** Focus group topic guide

**Additional file 2:** Additional quotes supporting the findings

**Additional file 3:** Summary of views on contextual influences on DP and POC-CRPT

**Additional file 4:** Reporting checklist

## Data Availability

The dataset analysed during this study is available from the corresponding author on reasonable request.

## Abbreviations

COPD: Chronic obstructive pulmonary disease
CRN: Clinical research network
CRP: C-reactive protein
DPs: Delayed antibiotic prescriptions
GP: General practitioner
HCA: Healthcare assistant
NICE: National Institute for Health and Care Excellence
POC-CRPT: Point-of-care C-reactive protein testing
RTIs: Respiratory tract infections
STAR-PU: Specific therapeutic group age-sex related prescribing unit
UK: United Kingdom
