# Supplementary material for: Implementing interventions to reduce antibiotic use: a qualitative study in high-prescribing practices
Source: BMC Fam Pract. 2021 Jan 23;22:25. doi: 10.1186/s12875-021-01371-6 (PMC7825381; doi:10.1186/s12875-021-01371-6)
Supplement: Supplementary file 1 — Additional file 1: Focus group topic guide [file 12875_2021_1371_MOESM1_ESM.pdf]

Additional File 1. Focus group topic guide

1. **Introduction / making prescribing decisions**
    a. How do you make a decision whether a patient needs an antibiotic / to prescribe an antibiotic?
    b. Is there anything in particular that would help you make this decision?

2. **Back-up / delayed antibiotic prescriptions (DP)**
    a. Introduce the NICE definition of DP: "one that is given in a way to delay the use of a medicine (usually an antibiotic), and with advice to only use it if symptoms worsen or don't improve within a specified time. The prescription may be given during the consultation (which may be a post-dated prescription) or left at an agreed location for collection at a later date".
    b. How does this relate to your own understanding of back-up-delayed prescriptions?
    c. Could you tell me about how you use back-up/DP in your practice?
    d. How do you explain back-up/DP to patients?
    e. How do you think back-up/delayed prescribing might influence antibiotic prescribing rates in your practice?
    f. How do you record use of back-up/DP? / How do you monitor use of back-up/DP?
    g. How do you think using back-up/DP could be improved or made easier in your practice?
    h. What are the (other) advantages / benefits of using back-up/DP?
    i. What are the (other) disadvantages / problems with using back-up/DP?

3. **Point of care CRP testing (POC—CRTP)**
    a. Have you had access to point-of-care CRP testing machines in your practice? Has anyone had experience of using POC CRP tests? (E.g. in other practices)
    b. Introduce / describe three different types of POC-CRPT: the Alere Afinion analyser, SureScreen, and Febridx.
    c. What type of test would you prefer and why?
    d. What are your views on introducing CRP tests in your practice?
    e. How would you use this CRP tests in your surgery?
    f. How do you think CRP testing might influence antibiotic prescribing rates in your practice?
    g. What are the (any other) advantages / benefits of using CRP tests?
    h. What are the (any other) disadvantages / problems with using CRP tests?
    i. How do you think POC CRP testing could be funded?

4. **Communication and other AMS strategies**
    a. How is information communicated in your practice? For example, guidelines updates, prescribing data or any changes / new initiatives.
    b. Are there any other initiatives or strategies that you have used to improve antibiotic prescribing?
    c. Are there any other initiatives or strategies that you would like, or are planning, to try out in your practice to further improve and reduce antibiotic prescribing?
    d. Is there anything else that you would like to mention that we haven't talked about?
