# Supplementary material for: Implementing interventions to reduce antibiotic use: a qualitative study in high-prescribing practices
Source: BMC Fam Pract. 2021 Jan 23;22:25. doi: 10.1186/s12875-021-01371-6 (PMC7825381; doi:10.1186/s12875-021-01371-6)
Supplement: Supplementary file 2 — Additional file 2: Additional quotes supporting the findings [file 12875_2021_1371_MOESM2_ESM.pdf]

Additional File 2. Additional quotes illustrating the findings

1. Compatibility of strategies with clinical role and experience

- **Clinical assessment and judgement at the core of clinical roles in general practice, e.g.:**

It would be a very clinical decision so each patient would be looked at holistically in their own context and then we would listen to the history so what the patient was telling us about what was wrong with them. We'd then do a clinical examination to aid that history taking (…) We don't often do blood tests before we prescribe. We would do if we thought it was relevant and we would certainly do a blood test to investigate things but it's not normal clinical pattern to wait for that. Then sometimes we do prescribe antibiotics without clinical signs if it's a long-term patient and they're susceptible to something and we're worried about something else in their history. So if they're immunocompromised for example or starting chemotherapy or something like that, then we may well issue a prescription but without the evidence of a clinical infection but we know it's in the best interest going forward. That's how we normally do it. [GP, FG1]

- **Strategies useful when clinically uncertain, e.g.:**

I'll say 'well I don't know. You're borderline. You could go either way, have these in case but don't take them yet'. So I suppose that might be called as me safety netting. [GP, FG3]

It's the ones that are borderline and maybe the ones where I'm thinking 'shall I give you a delayed prescription? Let's just test you' (…) there may be a few, I'm not sure there would be that many (…) I can't see it making a massive difference, but yeah in a few. [GP, FG3]

I'll probably use [POC-CRPT] somewhere in the middle with patients when you're not sure basically. If you are sure that you're going to give antibiotics then you will give or not. [GP, FG8]

- **Perceived incompatibility of POC-CRPT with clinical role in general practice, e.g.:**

GP1: I think most patients that would have a high CRP have other clinical signs as well; so I think it's few and far between where you get a really high CRP out of the blue and the patient we're not thinking he's unwell for another reason. (…) A lot of the time clinically a CRP wouldn't alter my judgement. I would see them and if they've got clinical signs or symptoms or their history, I would start antibiotics or admit them anyway.

GP2: A lot of certainly how [GP1] and I would have been trained historically is based on what you find examining the patient and their history and their overall clinical state within the context of their current or previous medical history… certainly how I was trained was that you're doing a blood test looking for something, not as a screening for something. I would agree that actually a negative CRP is not going to change my mind… (…) I don't think doing it would necessarily change how I would manage things. It's primarily based on the history and the examination and the background of the patient. One of my trainers always used to say 'treat the patient, not the numbers' and yes, numbers are important sometimes but in the wider context of the patient. [FG1]

How doctors work is we've almost certainly decided what the diagnosis is within 30 seconds of you opening your mouth, from history alone, and examination might add a bit, everything else, tests don't

diagnose, certainly not CRP because it's such a broad test. Tests do not diagnose things, they just back up what we're already thinking. [GP, FG4]

Secondary care they can do [POC testing] every single day and it's so much easier. It's there, you decide. For us it's more like what you can see and this is just an additional aide, which we have no luxury to do it every day. [GP, FG6]

- **Strategies more useful to those less clinically experienced, e.g.:**

[Prescribing pharmacist] he's at the start of his clinical training and I think you do follow the rules and the regulations a bit more because of your lack of experience. [GP, FG1]

I think possibly if you had a trainee, would have very inexperienced doctors, [CRP testing] might be a nice way of doing it. Or if you've got advanced nurse practitioners or pharmacists or people that have just started out that want the reassurance of saying to patient. [GP, FG1]

I think it's difficult at the start of training in that you've not got that much experience and therefore it's important that the patient trust what you're telling them and you're more worried about making a mistake. I probably had a bigger range of ones that were in the middle and I thought should I be prescribing or not, and thought on balance probably not, but then I felt more comfortable having that safety net. I think as I've progressed in training I've not needed that anymore in my prescribing. (…) now I don't need that and it's more about the patient and things like is it a Friday, what are things going to be like over the weekend. It's thinking about it more from [patient's] perspective because I feel generally a bit more confident. [GP Trainee, FG5]

2. Strategies used as social tools to negotiate treatment and educate patients

- **Patient expectations as a major challenge; reducing antibiotics beyond GPs' control, e.g.:**

The biggest issue around antibiotic prescribing is not my own knowledge or confidence on when or when not to. It is when patients are expecting to be given antibiotics… I will have patients walking in and saying 'I've got a chest infection.' Well, no, you don't have a chest infection necessarily. What you have is a set of symptoms and then it's down to me to decide based on that whether you have a chest infection or not. [GP, FG1]

[Patients] have got unrealistic expectations. They want to get better quite quickly. […] that sometimes can be difficult but I don't know what the solution for that is. [GP, FG7]

It takes a lot of time and effort then and there's been a lot of [patients] that keep coming back and back and you're not getting anywhere, and then you can get quite frustrated that way as well… The other thing they can do is sometimes they shop around, so they go to different areas. Sometimes they will see us and we'll say, 'We haven't got any antibiotics.' Because we've got that many services available, they might use the GP Hub and go there that same day and they would prescribe it… Or they go to a walk-in… [GP, FG7]

Inevitably sometimes we do treat viral infections with antibiotics anyway. It's just something that happens unfortunately. [GP trainee, FG9]

- **Using DPs as a 'social tool' to negotiate treatment and educate patients, e.g.:**

Before that, it [DP] probably was providing a bit of a compromise. Whereas now I feel more confident in saying this is viral or this needs antibiotics.(….) I think it's difficult at the start of training in that you've not got that much experience and therefore it's important that the patient trust what you're telling them and you're more worried about making a mistake. I probably had a bigger range of ones that were in the middle and I thought should I be prescribing or not, and thought on balance probably not, but then I felt more comfortable having that safety net. [GP trainee, FG5]

That's probably my commonest scenario, that they don't want to leave my room without something and it's a compromise – possibly more so than my clinical feeling that this is a good idea. [GP, FG3]

GP1: I tend to use [DPs] in the people you just cannot convince that they don't need antibiotics. You can tell when you're talking to them that the shutters have gone up, and there's a big temptation as a doctor to want to do something (…) when in reality they don't need anything. It's just sometimes it's just the route of least resistance, they're going away with a prize, they've got their little bit of paper, but most of the time, as far as I can… most of the time they either need antibiotics or they don't. […]

GP2: It's used as a way to make our life a little bit easier. (…) It makes it easier because you've come up with a trade-off, you're using it as a trade-off and if you're saying, 'come on, give my way a bit of a chance, let's see how it goes, and if you do what I said, all the stuff like steam and all that kind of stuff, then it will help', and most of the time you'll see that they start improving. You say it takes seven to ten days for it to get better, and if then in a few days they're starting to feel a little bit better, they say, 'okay, we're on the right track', and that's when they don't come in for the antibiotics. (…) The advantages are that in those patients that are going to get worse, it's another option, rather than wasting another appointment.

GP1: I suppose it leads to improved patient satisfaction, because they feel they're not being fobbed off. I have a very simple rule, which an older GP taught me, and it's, even if it's just a bit of advice, everybody leaves with a prize. They've made the effort to come and see a doctor, give them a bit of advice or something like that, or even a prescription or a form for physio or something like that, it's the key to not getting complaints. Everybody gets a prize, even if it's just a bit of written paper or something like that. [FG4]

Certainly from a patient perspective, they feel that they've been heard and you've understood how concerned they are and you've listened to what they've had to say (…) It helps cement that equal footing within the consultation… it's taking into account that your patient has views and considerations that you need to take into account, so it might help cement that they think, 'I am responsible enough to make my own decision about this thing and I will listen to their advice,' and sometimes I think that can help, going forward, as well. [GP, FG7]

- **Using POC-CRPT as a 'social tool' to negotiate treatment and educate patients, e.g.:**

Pharmacist: [Using POC-CRPT] Definitely when you justify not issuing a prescription. There you go, this is your result. This is the guidance.
Business Partner: Yes, it's not me. It's science. [FG2]

…where the patient is really pushing for the antibiotic that you don't think they need and you could prove it – it would be annoying if it came back positive. [GP, FG3]

GP: Near patient testing would also be quite useful because it's nice to have something objective. Most of what we make our diagnosis on is subjective history and examination, but if you've got an objective thing where the CRP is raised, then you can justify what you're doing more, which is increasingly from a medico-legal point of view, increasingly important.
Nurse: And if it's not raised, you've got the proof to justify it to the patient.
GP: Yeah, exactly.
[...]
GP: If you got a printout, you can give [patients] a copy, it's a prize, they've had a test. They think tests are how we do medicine, and they're not. [FG4]

GP1: I think it's massively helpful for the patients learning. If you can say 'Look, we've done this test and that would suggest that you haven't got an acute bacterial infection' because I think a lot of this is about patients learning that those symptoms are viral and will resolve.
GP4: It virtually excludes it there and then if they've got a very low CRP so you can persuade the patient apart from the fact they've got nothing else, this is what it tells me. [FG5]

I could also use it on these frequent offenders who come in saying 'I want antibiotics'. So if you keep testing, this is a waste actually, but if you show them it's not this and it's not 100 and that convinces them in some ways psychologically not to get the antibiotic. [GP, FG8]

...those demanding patients... especially if they don't take your education and they just want antibiotics so that's where that objective comes in and 'Ah hah!' They don't have a case. [GP, FG9]

I think for me [POC-CRPT] is more to deny patients antibiotics to be honest more than deciding on antibiotics or not, especially with the cold and flu symptoms. It's easier to say no but if I'm to decide antibiotics that's my clinical decision. [GP, FG9]

- **Unintended consequences of using POC-CRPT as a 'social tool', e.g.:**

Pharmacist: My fear is if this is available I think people will say I'll go and try one of those [POC-CRPT] out. [...]
GP: It may end up being part of a lot of people's routine consultations which it shouldn't be really but I think that may cause an issue because obviously it costs as well every time you do it. [...]
Pharmacist: It can medicalise the common cold almost if you know what I mean. [...] It's one of those that it's adding in a level of diagnostic that doesn't need to be there that maybe perhaps further stripping people of common sense. [FG2]

GP1: The disadvantage is that we would get so that we'd be doing them a lot and what would happen in reality is people would be asking for them... They'd be using those appointments... so more healthcare appointments. 'Can you just check the CRP?'
GP5: And if it takes even three minutes we'd be sending them down to your room saying 'I've got four people waiting. Can you do me a CRP?' So your workload would increase. [FG5]

3. Ambiguities about usefulness and impact of strategies

- **Ambiguity about situations and patients when DPs should or should not be used, e.g.:**

GP: I wouldn't normally do a deferred script to a child.
Pharmacist: No.
GP: Someone that's at the risk of something like neutropenia sepsis or people under chemotherapy and things like that. They have to see a doctor if they've got a temperature or feel unwell. [...]
Pharmacist: I've done two [DP]. [...] One of them the patient had just finished chemotherapy I think and the other one was a child who wouldn't be examined properly. [FG2]

[DP is] for elderly patients who have managed to come and see us on a Friday but assume they get worse on the weekend, for them to travel from there or to the nearest walk-in centre (…) So just to reduce that hassle sometimes and anxiety. [GP, FG6]

If it's a bank holiday or they're going away, even if it's somewhere local, then I might say to them, 'Don't go and get it. Just hang on to it and see how they get on.' That's generally when we offer delayed prescribing – it's when they don't have access to come to us. Otherwise I would say to them, 'Give us a call back and we'll see you again or we'll speak to you again.' [GP, FG7]

If it's for a child or something like that, then I'd rather review them. If you have a compromised patient or a diabetic patient, then I might issue the script because I know they are at a higher risk so it all depends. It's not a fixed thing. It just depends on the individual. If it's a Friday then you have to issue it because Saturday and Sunday the surgery is closed. [FG8, GP]

- **Lack of feedback on, and perceptions of how, patients use DPs, e.g.:**

I'd like to see how many of those who are actually collecting them. It would be nice to be able to see whether they were dispensed or not because then you might not see the patient again for a year and you might forget to say that last time I gave you a delayed prescription. [GP, FG9]

When you give them the prescription and it's post-dated, you worry that they would cash it in anyway and keep it back home. Sometimes we go to people's houses to do home visits and then you open the drawer up and there's boxes of amoxicillin... half of them, they've been had and then half haven't. [GP, FG7]

- **Lack of set ways of doing DPs and uncertainties about DP formats, e.g.:**

We're tried a couple of different ways and I think everyone does it a bit different actually. I don't think we have any set ways. [GP, FG2]

GP1: I'm not sure what others are doing. I think post-dated is how I work, collecting at a nominated site can increase patients' anxiety of collecting it on the day, if possible, workload for the admin staff or to the pharmacy, whichever way. So I just post-date it and I think that works pretty good.
GP2: So yeah, handing the prescription to the patient but they can't get the prescription issued until the date you've specified on it. [...] I like to print out a post-dated prescription because actually giving them something in their hand to go away with gives them a sense that something's happening. [FG6]

...electronic prescribing, I tend to avoid that because (...) I don't know whether it becomes a problem for that patient if they don't go and pick it up. I don't really know what happens to the payment side of it (...) That's why I've stuck to giving it to them and saying, 'Don't go and get it,' and hope that they don't. [GP, FG7]

- **Ambiguity about situations/patients when POC-CRPT might be useful or not, e.g.:**

The patients that I would use it for, it's thinking would I admit this person or not, rather than thinking am I going to give antibiotics. I don't think I'd use it that much. [GP trainee, FG5]

I think if you've assessed the patient and it's your standard, youngish patient with sore throat whatever (…) then I'd believe the test and I'd treat them or not treat them. (…) if I want to prescribe I don't think I'd even do the test… I don't feel as though I need that backup with the test to be honest. Whereas it would be 'I'm borderline and I'm not sure'. [GP, FG3]

GP2: I'm just thinking now… Should we be using it specifically for patients where we've decided they've got a pneumonia but they don't need to be admitted and then we do a base line CRP and we check that…
GP4: So you've already made the decision.
GP2: …48 hours their CRP has come down with the antibiotics you've given them. It might be a useful way of reassuring yourself that you can keep them out of hospital. [FG6]

GP1: Would it help with patients like COPD who has exacerbation but not productive, you know not producing [copious flem?] and sometimes we say 'Start with steroids, do the antibiotics if your flem goes over or if you produce – you know, in those cases would you do those? I'm just thinking.
GP2: I think once you've started using steroids, it can make the CRP interpretation more difficult. [FG6]

Would you take it on a home visit if you thought a patient was sick? Or take it on a home visit in case you got there and the patient was not making a lot of sense. We often find you have a patient at home who doesn't make a lot of sense and you don't know whether that was how they were anyway or it's because they didn't take their tablets. (….) 'Cause if you do send them in, they usually do do their CRP and it is usually in the 90s, isn't it, when you admit an elderly patient who's not left their bed for a few days. That might be a possible use for it, perhaps. [GP, FG7]

- **Uncertainty about evidence, acting on results and medico-legal consequences of POC-CRPT, e.g.:**

I don't know enough about how specific CRP is to a bacterial infection really off the top of my head because there are a number of reasons why CRP can go up, so yes it is an acute reaction protein but we know that it increases with age and we know that, you know, the patient might have PMR [Polymyalgia Rheumatica]… I don't know enough about it to be confident that a CRP of more than 20 definitely means that this patient needs antibiotics. [GP, FG3]

Is it suitable for all infections? That's what we need to know more about, the indications. For consistency, a bit like the prescription, we all need to work for the same guidance on that. [GP, FG5]

How accurate are these [POC-CRPT]? Do we know that these are, have these been validated against other tests? [GP, FG5]

CRP of 40 or CRP of 50, that could be anything. It could be they've got rheumatoid arthritis at the same time with it or IBS. [GP, FG1]

I would worry that by doing it and then getting a result that I wasn't necessarily expecting, I would then feel obliged to prescribe something because otherwise I'm not acting on an abnormal result. (…)

I'm quite wary of doing tests to reassure myself that there's nothing going on. We all do it sometimes but I suppose, as I say, was taught don't do things unless you're looking for a particular reason or you think something is going to be abnormal because if you're just doing it to reassure yourself sometimes you'll find you get the result you don't want and then you've got to decide how you're going to proceed from there. [GP, FG1]

- **Mixed views about impact of POC-CRPT on antibiotic prescribing, e.g.:**

I think most patients that would have a high CRP have other clinical signs as well; so I think it's few and far between where you get a really high CRP out of the blue and the patient we're not thinking he's unwell for another reason. (...) I think a lot of the time clinically a CRP wouldn't alter my judgement. I would see them and if they've got clinical signs or symptoms or their history, I would start antibiotics or admit them anyway. [GP, FG1]

There would be a reduction but I don't think it would a dramatic reduction 'cause I think it will still be clinical. [GP, FG6]

Is it gonna change our management in our day to day care? Not that much. So is there any incentive for us to do that in the first place? [GP, FG6]

Supposing, after doing all these grey area patients, your antibiotic prescribing went up, what would you do then? [GP, FG7]

4. Influence of context on use of strategies

- **Practice context/characteristics influencing DP use, e.g.:**

We have quite a lot of access here, so they tend to phone back rather than the delayed. [GP, FG7]

GP: Because we're a small practice I think we're reasonably able to keep track of who is requesting antibiotics frequently and generally we don't have much of an issue with patient access. (...)
Nurse: We get to see [patients] over and over again and get to know, recognise whether they do have an infection that needs treating or not. Because we know them, we can step in earlier which might seem that clinically they might not have all of those markers that you would give antibiotics for but we've seen it before going in that direction so it might seem they started antibiotics quite early before they did the swab but you've seen it before because they've come in chronically with infection. [FG1]

The vast majority of our patients, again because of us being rural, we dispense the medication to because they live too far away from a pharmacy to do it the other way, which in some sense does make things a little bit trickier because we don't physically give people a prescription... [GP, FG1]

GP2: We have a very deprived area. [Patients] are not debating in their minds about I'm going to come in five days and this is the signs to come and check in with them. They want something now or they want something to be done. We might sit and talk about everything but it doesn't really get through. They panic more so if you do a delayed script it's as good as not doing one so if you get them back that gives more reassurance. [...]
GP1: We do have an awful lot of trainees here and we have a lot of foundation year doctors here now so they're only coming for four months, so you've just about got them into the zone of: 'we don't always prescribe penicillin you know', and then they've left and a new lot have come; so I think

we tended to think that it's the number of trainees and the ease of access and the area with a lot of COPD and things so…

GP2: Yeah very high prevalence of chronic diseases. [FG3]

- **Practice context and practical factors influencing perceptions of feasibility of POC-CRPT, e.g.:**

If you came across your patient that you weren't 100% sure of, how to then get another member of the team to do it? They're all busy, like you'd have to have one person on stand-by. It's not practical, it's just not going to work. [GP, FG3]

GP1: …by the time we've done our history, examination and made the decision that mm, is it or isn't it? I'm going to do the test, then that's too much extra time added on then. […]

GP2: …if you didn't have a clinical fridge in your room, then you had to come up to central storage fridge, wait for that consultation to finish in order to go into the room to get the stuff that you needed, to then go back to the patient. It's too, it's too much faff. General practice doesn't work like that. It might work well in something like A&E where you can park a patient as it were, and say 'we're going to go and run a test. I'll be back in 20 minutes'. [FG3]

GP5: I don't think we'd do it in our appointments… because that's an extra three or four minutes on a ten minute appointment.

GP1: I think the logistics of it, the logistics of the building are by the time we've then taken a patient, and certainly the analyser, because we'd have to go to for analyser wouldn't we, I don't think it would work.

GP4: And the other thing is you're waiting for the time for the result.

Nurse: I can't see the benefit of that expense.

GP4: So technically it's better for us to crack on with our surgery because we can't wait another five minutes for that result to come through.

GP1: To be fair, we think that we're relatively modern but we still have quite an old fashioned style of consulting and we still get quite a lot of acute things coming to GPs where a lot of surgeries would have handed that to nurse practitioners. If we got to a point where actually all the acute on the day stuff was nurse practitioner led and they were down in the nursing room with that sort of thing there, that's a different ball game. I think coming from doctors to nurses I think the flow would be hard just because of the way our surgery is set up. [FG5]

GP1: I think it would probably be helpful to have two or three trained staff, so it would probably be us directing the patient to the nurses, for example. Our nurses are very under pressure at the moment with appointments, so it's how you would build that in and having an agreement with them (…)

Nurse: Fridge space, especially at flu season, 'cause we've got three fridges here, all full of immunisations, so fridge space is a big thing. [FG7]

- **Wanting to try POC-CRPT but requiring funding for it, e.g.:**

We should be doing it. […] I think we have to now look at the world, the way the world is… not just our practice and not just the UK, which is crap, it's just everywhere else. We are way behind everyone else and what's happening with everyone else is they're getting more and more tests… so we need to kind of keep up with that… So if the world is doing it, you've got to do it. [GP, FG4]

Practices are not actually businesses. We're told we are and we've got all the responsibilities of businesses without any of the rights of the business. We can't earn any more money by doing better work. We just earn a set fee. [POC-CRPT] needs to be funded centrally because it's the NHS, not the [practice name] service. It's a central saving. The prescribing never hits us so if the prescribing goes up or down, it doesn't bother us because it's not our budget. We don't fund our budget; it's funded centrally so if the central people are feeling the benefits, the central people need to do the investment as well… [Business Partner, FG2]
