# Supplementary material for: Implementing interventions to reduce antibiotic use: a qualitative study in high-prescribing practices
Source: BMC Fam Pract. 2021 Jan 23;22:25. doi: 10.1186/s12875-021-01371-6 (PMC7825381; doi:10.1186/s12875-021-01371-6)
Supplement: Supplementary file 3 — Additional file 3: Summary of views on contextual influences on DP and POC-CRPT [file 12875_2021_1371_MOESM3_ESM.pdf]

Additional File 3. Summary of views on contextual influences on DP and POC-CRPT

Table S1. Practice characteristics and summary of views on practice and practical influences on DP and POC-CRPT use

Focus group	Urban/ rural	Deprivation (decile) [a]	Onsite dispensary / pharmacy?	FG participants [b]	Reported impact of practice characteristics & practicalities on DP use	Reported impact of practice characteristics & practicalities on POC-CRPT use
FG1	Rural (village)	Medium (5)	Dispensary	2 GPs, Nurse, HCA, Practice Manager	• Good access, telephone triage & small practice (knowing patients well) – prefer to re-consult than use DPs<br>• Rural with dispensary – patients collect antibiotics from the dispensary after consulting, reducing the need to return to pick them up (distance & transport make it difficult); GPs advise patients to wait before taking antibiotics rather than giving DP<br>• Medium deprivation – patients described as 'well educated' & 'well informed' so it is easier to manage without antibiotics/DP	• Rural – limited ability to send blood samples to hospital and get results quickly (POC-CRPT may help in such situations)<br>• Practical – the analyser would likely be in nurses' room<br>• Staff – GPs would ask nurses or HCAs to do the tests for them; nurses & HCAs are busy with their own patients so could only do the tests in certain situations<br>• Time seen as the biggest practical barrier
FG2	Rural (town and fringe)	Medium (5)	Pharmacy	GP, Pharmacist Prescriber, Business Partner	• No set ways of using DP<br>• Practical – had a box for DPs at the reception but stopped using it as it was unhelpful because patients could not access DPs when the practice was closed	• *Practice had received an analyser from the company but had no funding for CRP cartridges so had not used it*<br>• Time and cost seen as the biggest practical barriers<br>• Triage – perhaps could do the tests at the reception before seeing a GP
FG3	Urban (major conurbation)	High (2)	No	2 GPs, Prescribing Clerk, Practice Manager	• Good access & telephone triage – patients can re-consult easily rather than using DPs, but DPs might reduce consultations<br>• Practical – had a box for DPs at the reception but stopped using it as it was unhelpful because they 'had a lot of	• *Practice had an analyser and received training as part of a research study, but had not used it on patients as the study closed before they started recruiting*<br>• Time, cost & 'too much faff' seen as the main barriers (unless the analyser was in

					patients just coming and trying to collect them', 'being quite confrontational', 'challenging the receptionists' and 'then the receptionists were having to make the decision to book the patient back in with the doctor'	all consulting rooms which would be too expensive) – POC-CRPT seen as impractical, not fitting in GP consultations ('general practice doesn't work like this')<br>• Telephone triage could be used to book patients for the test<br>• Staff: HCA could do the tests; no nurse prescribers or pharmacists who could test
FG4	Rural (town and fringe)	Medium (4)	Dispensary	2 GPs, Nurse, Practice Manager	• Rural – prefer to ask patients to call if their symptoms worsen, then antibiotics can be issued and collected from the dispensary (rather than using DPs)	• Practical – the analyser would likely be in the nurses' room<br>• Staff – GPs would likely ask nurses to do the tests; nurse triage could be used for nurses to test and direct patients with high results to GPs
FG5	Rural (town and fringe)	Low (7)	Independent pharmacy on site	3 GPs, 2 GP Trainees, Nurse Prescriber, Practice Manager	• No set ways of using DP<br>• Rural – 'logistical difficulty' of asking patients to return to pick up DPs so prefer to give DPs & ask patients to wait before using them rather than come back	• Time & 'the logistics of the building' (location of the analyser) seen as the main barriers to use<br>• Staff – testing likely to be led by the Nurse Prescriber (NP) or HCAs, but it would increase and disrupt their workload; as NP role expands she could test more<br>• Portable tests could be used by the community matron on home visits
FG6	Urban (major conurbation)	High (3)	No	4 GPs, Medicines Coordinator	• No set ways of using DP<br>• Extended access – patients can consult more easily<br>• Practical – asking patients to collect DPs from the practice avoided due to 'workload for the admin staff'	• Practical – the analyser could be in the treatment room and used by GPs<br>• Staff – nurses are not prescribers so would not use POC-CRPT, unless asked to do it for GPs but it would disrupt their workflow
FG7	Urban (major conurbation)	High (2)	No	3 GPs, Nurse	• Good access, telephone triage & extended access – prefer to ask patients to call or re-consult than use DPs	• Patient characteristics – 'In the educated population they wouldn't really care what their CRP was' as they could rest until they get better

					• High deprivation – patients described as 'deprived community' who often consult early in an illness & expect antibiotics; thus, important to educate them with advice & leaflets (rather than using DPs)	• High turn-over of their patients and staff – harder for patients to trust the doctor so POC-CRPT could help reassure patients<br>• Practical – cost, storage, training and the 'logistics' seen as the main barriers to use<br>• Staff – GPs would ask nurses to do the tests
FG8	Urban (major conurbation)	High (2)	No	6 GPs, 1 GP Trainee, 2 Nurses, Practice Manager, Deputy Practice Manager	• Good access & out-of-hours – prefer to re-consult than use DPs<br>• High deprivation – 'very deprived area' with patients ('from abroad') described as expecting antibiotics & not understanding DPs; thus, prefer not to use DPs as patients may not understand, see it as a conflicting message, use it inappropriately	• Practical – GPs might use the portable tests if they are in every consulting room<br>• Time seen as the main barrier to use<br>• Staff – nurses might do the tests if asked by GPs, but they are not prescribers so would not use them with their own patients
FG9	Urban (major conurbation)	High (1)	No	2 GPs, 2 GP Trainees, HCA, 2 Receptionists	• Limited access – if they had more appointment availability, they would prefer to re-consult than give DPs	• Practical – the analyser likely to be in the emergency or treatment room<br>• Time seen as the main barrier to use<br>• Staff – GPs would ask nurses or HCAs to do the tests for them; perhaps could train the receptionists to test while the patients are waiting (after agreeing a protocol on when to test) or test in specific 'cough and cold' clinics

[a] *Index of multiple deprivation decile.*

[b] *HCA – GP – General Practitioner, Healthcare Assistant (non-prescriber), Nurse – Practice Nurse (non-prescribe*
