# Supplementary material for: Implementing interventions to reduce antibiotic use: a qualitative study in high-prescribing practices
Source: BMC Fam Pract. 2021 Jan 23;22:25. doi: 10.1186/s12875-021-01371-6 (PMC7825381; doi:10.1186/s12875-021-01371-6)
Supplement: Supplementary file 4 — Additional file 4: Reporting checklist [file 12875_2021_1371_MOESM4_ESM.pdf]

Additional File 4. Reporting checklist

The checklist:
O'Brien BC, Harris IB, Beckman TJ, Reed DA, Cook DA. Standards for Reporting Qualitative Research: A Synthesis of Recommendations. Acad Med. 2014;89(9):1245–51.

No.	Topic	Page number (and/or additional details)
**Title and abstract**		
S1	Title	1
S2	Abstract	2
**Introduction**		
S3	Problem formulation	4-5 (Introduction)
S4	Purpose or research question	5
**Methods**		
S5	Qualitative approach and research paradigm	5-6: qualitative study with semi-structured focus groups; thematic analysis, taking an inductive, realist approach to analysis and identifying themes on a semantic level (as outlined by Braun and Clarke, 2006).<br>• No specific theory was used to guide the study.<br>• Rationale:<br>○ Focus groups were used so that participants shared, discussed, and addressed similarities and differences in their views/experiences and prompted each other to consider different perspectives and arguments.<br>○ Inductive thematic analysis was used to identify the 'higher-level' types of factors and considerations influencing professionals' views on the implementation and usefulness of DPs and POC-CRPT as strategies to optimise antibiotic use.
S6	Researcher characteristics and reflexivity	5-6: Focus groups were conducted by AB & AC – female non-clinical health sociologists with PhDs and experience in qualitative health research, but without specific clinical knowledge or experience of DPs/POC-CRPT which helped limit own assumptions and presuppositions. Participants and researchers had not known each other prior to study.<br><br>Coding of transcripts was conducted by two researchers (AC & AB, health sociologists), with a selection of transcripts double-coded by STC (health psychologist and senior qualitative researcher with knowledge and experience of researching interventions to optimise antibiotic prescribing) and ED (psychology student). The analysis was discussed throughout with MM (pharmacist and senior researcher experienced in researching interventions to optimise antibiotic prescribing). The themes and findings were discussed also with the wider STEP-UP study team comprising experienced researchers with multidisciplinary background (GPs, epidemiologists, behavioural economists) and topic-specific expertise. The multidisciplinary team helped ensure that any potential bias was minimised.

S7	Context	5: For practical reasons, focus groups were conducted in each participating practice (in practice's meeting rooms, mostly during lunchtimes or afternoons).
S8	Sampling strategy	5
S9	Ethical issues pertaining to human subjects	5 and the 'Ethical Approval' section
S10	Data collection methods	5-6
S11	Data collection instruments and technologies	5-6
S12	Units of study	6: Units of analysis were practices (focus groups) – their characteristics are reported in Table 1. Individual participants were characterised by their professional roles.
S13	Data processing	6
S14	Data analysis	6
S15	Techniques to enhance trustworthiness	• Researcher triangulation: data collection and analysis were conducted by multiple experienced qualitative researchers, transcripts were double-coded and the analysis discussed with a multidisciplinary team.<br>• Audit trail: detailed notes were kept throughout the study: prior to data collection (e.g., practice and participant characteristics), during the data collection (helping to identify individual speakers in the transcripts), immediately after the focus groups (to capture researchers' observations, anything discussed when the audio recording was off), and throughout the analysis (e.g., researchers' reflections and discussions).
**Results / findings**		
S16	Synthesis and interpretation	'Results' section
S17	Links to empirical data	Selected quotes illustrating the findings are reported in the Results section, with additional quotes illustrating and supporting the findings reported in Boxes 2 and 3, and in Supplementary Document 2.
**Discussion**		
S18	Integration with prior work, implications, transferability and contribution(s) to the field	'Comparison with existing literature' section
S19	Limitations	'Strengths and limitations' section
**Other**		
S20	Conflicts of interest	'Competing interests' section
S21	Funding	'Funding' section
